# Genetic decline, restoration and rescue of an isolated ungulate population

**DOI:** 10.1111/eva.12706

**Published:** 2018-11-27

**Authors:** Marc‐Antoine Poirier, David W. Coltman, Fanie Pelletier, Jon Jorgenson, Marco Festa‐Bianchet

**Affiliations:** ^1^ Département de Biologie Université de Sherbrooke Sherbrooke Québec Canada; ^2^ Centre d’Études Nordiques (CEN) Université Laval Quebec City Québec Canada; ^3^ Department of Biological Sciences University of Alberta Edmonton Alberta Canada; ^4^ Alberta Environment and Parks Canmore Alberta Canada

**Keywords:** bighorn sheep (*Ovis canadensis*), bottleneck, conservation management, fitness, genetic rescue, inbreeding depression, translocation

## Abstract

Isolation of small populations is expected to reduce fitness through inbreeding and loss of genetic variation, impeding population growth and compromising population persistence. Species with long generation time are the least likely to be rescued by evolution alone. Management interventions that maintain or restore genetic variation to assure population viability are consequently of significant importance. We investigated, over 27 years, the genetic and demographic consequences of a demographic bottleneck followed by artificial supplementation in an isolated population of bighorn sheep (*Ovis canadensis*). Based on a long‐term pedigree and individual monitoring, we documented the genetic decline, restoration and rescue of the population. Microsatellite analyses revealed that the demographic bottleneck reduced expected heterozygosity and allelic diversity by 6.2% and 11.3%, respectively, over two generations. Following supplementation, first‐generation admixed lambs were 6.4% heavier at weaning and had 28.3% higher survival to 1 year compared to lambs of endemic ancestry. Expected heterozygosity and allelic diversity increased by 4.6% and 14.3% after two generations through new alleles contributed by translocated individuals. We found no evidence for outbreeding depression and did not see immediate evidence of swamping of local genes. Rapid intervention following the demographic bottleneck allowed the genetic restoration and rescue of this bighorn sheep population, likely preventing further losses at both the genetic and demographic levels. Our results provide further empirical evidence that translocation can be used to reduce inbreeding depression in nature and has the potential to mitigate the effect of human‐driven environmental changes on wild populations.

## 1 INTRODUCTION

Theories of genetic drift predict that small and isolated populations will suffer decreased genetic diversity and increased inbreeding over time, eventually compromising population persistence (Charpentier et al., 2005; Da Silva et al., 2009; Grueber, Wallis, & Jamieson, 2008). Inbreeding depression, reduced fitness of offspring born from matings among relatives, is ubiquitous in small populations and has been documented in many species (Charlesworth & Willis, 2009; Hedrick & Garcia‐Dorado, 2016; Keller & Waller, 2002). Without immigration, small populations affected by inbreeding depression are at increased risk of extinction (Saccheri et al., 1998). For many natural populations, however, natural or human‐caused isolation prevents gene flow and therefore reduces persistence probability (Broquet et al., 2010; Pavlova et al., 2017). In addition to isolation and low population size, demographic bottlenecks, rapid and persistent declines in population size, are important causes of inbreeding and loss of genetic variability (Broquet et al., 2010; Nei, Maruyama, & Chakraborty, 1975). Small populations with low genetic diversity are also expected to be less likely to adapt to environmental changes due to low evolutionary potential (Vander Wal, Garant, Festa‐Bianchet, & Pelletier, 2012), further increasing risk of extinction. Given widespread habitat loss, barriers to dispersal and population declines, especially of large mammals (Schipper et al., 2008), there is an urgent need for empirical data to guide management and conservation interventions seeking to maintain or restore genetic variation, and assure population viability.

Translocation of animals is increasingly used to reinforce populations of conservation concern, but its application as a technique to provide artificial gene flow in conservation biology and wildlife management remains rare and poorly documented (Ralls et al., 2018; Seddon, Armstrong, & Maloney, 2007). Translocations may seek two distinct but not mutually exclusive genetic outcomes. First, genetic restoration occurs when genetic diversity is increased by the addition of new alleles and changes in allelic frequencies (Whiteley, Fitzpatrick, Funk, & Tallmon, 2015). Second, genetic rescue occurs when population growth, inferred from some demographic vital rate or measured directly, is increased through reversal of inbreeding depression (Tallmon, Luikart, & Waples, 2004). To date, genetic restoration and rescue attempts through translocations remain uncommon (Frankham et al., 2017; Ralls et al., 2018). Yet, both genetic restoration and rescue have recently received empirical support as a means to mitigate genetic loss and inbreeding in the wild (Frankham, 2015; Whiteley et al., 2015). In contrast, there is much less evidence that translocations can contribute to evolutionary rescue, population recovery through genetic adaptation following environmental change (Carlson, Cunningham, & Westley, 2014). Nevertheless, natural or artificial immigration of conspecifics may facilitate evolutionary rescue by increasing the available genetic variation for selection to act on (Lenormand, 2002) or, although less likely in small populations, by increasing mutational opportunities through increased population size (Holt & Gomulkiewicz, 1997).

Translocations may also involve important risks to targeted populations. Potential spread of infectious disease and transmission of parasites are among the main concerns for conservation biologists and wildlife managers (Cunningham, 1996; Daszak, 2000). Moreover, outbreeding may not always be beneficial, particularly in the presence of local adaptations (Waller, 2015). Outbreeding depression, reduced fitness of offspring from matings between genetically divergent individuals, has been observed in wild populations and could render translocations counterproductive (Edmands, 2007). Outbreeding depression is generally associated with the genetic swamping of locally adapted variants by migrant alleles, fixed chromosomal differences between populations or the break‐up of co‐adapted gene complex (Edmands, 2007; Frankham et al., 2011; Lenormand, 2002). Translocation, as opposed to natural immigration, therefore presents greater risk to local populations through increased potential for maladaptation to environmental conditions (Edmands, 2007). Few studies, however, have documented in detail the genetic and demographic consequences of artificial population supplementation by monitoring all native and translocated individuals in a population (Frankham et al., 2017; Whiteley et al., 2015).

The bighorn sheep (*Ovis canadensis*) population of Ram Mountain, Alberta, is geographically isolated and immigration is rare. The population suffered a demographic bottleneck in 1992–2002 when a density‐dependent decline (Festa‐Bianchet, Gaillard, & Côté, 2003) was hastened by intense predation by cougars (*Puma concolor*) (Festa‐Bianchet, Coulson, Gaillard, Hogg, & Pelletier, 2006). It then failed to recover despite cessation of both predation and hunting pressure (Pigeon, Festa‐Bianchet, Coltman, & Pelletier, 2016) and stagnated at low numbers for several years. Rioux‐Paquette, Festa‐Bianchet, and Coltman (2011) found that inbred female lambs at Ram Mountain suffered a 40% decrease in overwinter survival. They found no evidence of inbreeding depression for male lambs and suggested that sex‐differential effects of inbreeding may be a general pattern in sexually dimorphic mammals because of sex‐biased maternal care or sexual differences in early development strategies (Rioux‐Paquette et al., 2011). In 2002–2007, the low and stagnating population size justified the translocation of bighorn sheep from another population to Ram Mountain, providing a rare opportunity to test the effectiveness of translocation for recovery in a wild population undergoing inbreeding depression.

Here, we explore how the deleterious consequences of a recent population bottleneck in this isolated population were at least partially overcome by supplementation from another, larger population. We first tested for loss of genetic diversity following the rapid decline in population size. Following translocations, we predicted an increase in genetic diversity through the addition of new alleles. Based on recent reviews of the beneficial effects of genetic rescue (Frankham, 2015, 2016 ), we expected a positive effect of outbreeding (i.e., admixture) on fitness‐related traits. Considering the sex‐differential consequences of inbreeding in this population (Rioux‐Paquette et al., 2011), we also predicted a greater effect of outbreeding on fitness of females than of male juveniles. We then explored whether differences in individual fitness affected the expected population growth rate and probability of persistence. Using detailed yearly demographic and genetic population properties, we present a precise description of both decline and recovery in a wild population of ungulates over four generations (27 years).

## 2 METHODS

### 2.1 Study population and translocations

Ram Mountain, Alberta (52°N, 115°W, elevation 1,080 to 2,170 m), is 30 km east of the Canadian Rockies. Due to its geographic isolation, immigration is rare. Since 1972, individually marked bighorn sheep have been monitored and captured 2–5 times per year (Jorgenson, Festa‐Bianchet, Gaillard, & Wishart, 1997). Since 1975, over 99% of resident adult sheep have been individually identifiable, so that the exact size and composition of the population have been precisely known each year (Jorgenson et al., 1997). The population declined from 232 sheep in 1991 to 40 in 2002, with only 21 adult females and nine adult males remaining. The population failed to recover despite the cessation of intense cougar predation in 2001 and stagnated at 40–45 individuals for 5 years (Festa‐Bianchet et al., 2006). Only 15 native adult ewes remained in 2007. To rescue the population, translocations were carried out in 2002–2007, when the resident population, including offspring of translocated sheep, ranged from 40 to 50 individuals. An additional nine sheep were translocated to Ram Mountain in 2015 (Poirier & Festa‐Bianchet, 2018) following high cougar predation in 2012–2013. This latter translocation was not included in our demographic and genetic analyses because none of those individuals had reproduced by 2016. Relocated sheep were captured in a large (over 500 sheep) population at Cadomin, Alberta (53°N, 117°W), 130 km northwest of Ram Mountain (Supporting Information Appendix S1), then moved by truck and helicopter in late winter, except for 6 rams that were translocated in November 2004 to allow them to take part in the rut. Translocated individuals were of sex‐age classes selected to promote short‐term demographic recovery, but within these sex‐age classes, they were chosen at random from the source population (Supporting Information Appendix S1). Translocated sheep were marked with ear tags and visual collars. In total, 26 sheep were translocated over 6 years. Of these, however, only 19 remained on the mountain by the time the next field season began late in the following May (Table 1). We do not know if the missing sheep had dispersed or died, and they were not considered in our analyses. Repeated captures and sightings of marked sheep allowed detailed monitoring of the Ram Mountain population throughout this study. At each capture, sheep were weighed and female reproductive status was assessed by inspecting the udder. At first capture, lambs were marked, sexed, weighed, and tissue samples were collected for DNA analyses.

**Table 1:** Sex, mean age at translocation and postrelease reproductive success of translocated sheep on Ram Mountain, Alberta, Canada (2002–2007)

Translocation	*n*	Sex	Age	RS^a^
2002	2	2 M	3.0	1 M
2005	12	6 M, 6 F	3.3	2 M, 1 F
2007	12	7 M, 5 F	1.0	3 M, 2 F

a Reproductive success; number and sex of those that produced at least one resident lamb.

### 2.2 Microsatellites and pedigree building

We obtained accurate measurements of the genetic decline and restoration of the Ram Mountain population following the demographic bottleneck and translocations, because nearly all individuals that survived to a few weeks of age were sampled. PCR amplification was performed at 26 ungulate‐derived microsatellite loci (Coltman, O'Donoghue, Hogg, & Festa‐Bianchet, 2005) for 585 (98%) sheep present on Ram Mountain in 1990–2016, or nearly four generations of bighorn sheep (Hamel, Gaillard, Festa‐Bianchet, & Côté, 2009). We excluded 14 individuals for which ≥20% of loci could not be amplified. For sheep included in genetic analyses, on average only 2.1% of loci could not be amplified. Yearly population estimates of genetic diversity were calculated using *GenAlEx* (version 6.5; Peakall & Smouse, 2012) (*N*_a_, *H*_O_, *H*_E_) and *FSTAT* (version 2.9.3; Goudet, 1995) (*A*_R_, *F*_IS_, *F*_ST_). We compared the genetic parameters of the translocated sheep with the native population (sheep born on Ram Mountain) using these measures. We also contrasted the population of native residents to a sub‐group of nonadmixed native residents (Ram Mountain ancestry only), to evaluate how the population benefited from admixture over time.

Maternal links were established from field observations of suckling behaviour. Paternal links were identified and maternal links confirmed from microsatellites, using *CERVUS* (version 3.0; Kalinowski, Taper, & Marshall, 2007) at a >95% confidence interval (Coltman, Festa‐Bianchet, Jorgenson, & Strobeck, 2002). Some individuals with unsampled fathers were identified as paternal half‐sibs using the software *COLONY* (version 2.0; Wang, 2004). Our pedigree allowed us to estimate the introgression of migrant alleles from translocated sheep at the individual level. We categorized each individual as “nonadmixed” (endemic Ram Mountain genotypes) or “admixed” (“F_1_”, “F_2_”, or “F_3_”) residents. The first admixed lamb was born in 2003. Thus, we only considered 2003–2016 for analyses of genetic rescue. Maternities and paternities were known for all 173 lambs born in 2003–2016. Of these, seven were F_3_ offspring that were excluded from genetic rescue analyses due to low sample size. During this period, nonresident males sired 39 (22%) resident lambs. These sires arrived at Ram Mountain for the rut, as is typical of bighorn sheep (Hogg, 2000). Nonresident sires, however, did not introduce new alleles and were therefore considered as residents to simplify analyses (Poirier,unpublished data). These sires likely came from Shunda Mountain, just across the North Saskatchewan River from Ram Mountain (*c*. 4 km). Movement of males between these two mountains has been documented, and both populations are considered the same genetic unit (Coltman, unpublished data).

### 2.3 Data collection and variables

To test for genetic rescue, we compared different fitness‐related traits of nonadmixed, F_1_ and F_2_ individuals born in 2003–2016. Of 166 lambs sampled, 87 survived to yearling age. For lambs, we considered four traits known to affect fitness: birthdate (Feder, Martin, Festa‐Bianchet, Bérubé, & Jorgenson, 2008), weaning mass, summer mass gain (Festa‐Bianchet, Jorgenson, Bérubé, Portier, & William, 1997) and survival (Gaillard, Festa‐Bianchet, & Yoccoz, 1998). Birthdates were determined through field observations of newborns with an estimated accuracy of ±5 days (Feder et al., 2008). Individuals were captured and weighed 1–4 times during their first summer and 2–7 times during their second summer (Jorgenson et al., 1997). Individual mass was adjusted to June 15 (lambs only) and September 15 (lambs and yearlings) using a mixed model based on individual recaptures (Martin & Pelletier, 2011). Lamb summer mass gain was calculated by subtracting mass adjusted to mid‐June from that adjusted to mid‐September. Survival to yearling age was determined in late May of the subsequent year through field observations and captures of individuals. For yearlings, we considered two fitness‐related traits: mass in September and survival to 2 years. At Ram Mountain, resighting probability is over 99% for ewes and yearlings (Jorgenson et al., 1997), and no sheep that disappeared as a lamb was ever sighted in a later year. We controlled for possible confounding effects of climate with the Pacific Decadal Oscillation (PDO; Mantua, Hare, Zhang, Wallace, & Francis, 1997). We used the mean PDO as a global climate index for summer (May–September), rut (November–December) and winter (November–March) seasons. We also controlled for possible confounding effects of maternal care on lamb traits. Mothers that weaned a lamb the previous year had higher energy expenditure than mothers who did not complete their previous lactation, which may affect the condition of their lambs the following year (Martin & Festa‐Bianchet, 2010). We therefore considered maternal mass in September and reproductive status the previous year in our analyses of fitness‐related lamb traits (Feder et al., 2008).

### 2.4 Statistical analysis

Statistical analyses were performed using the software *R* (version 3.3.1; R Development Core Team, 2015). Statistical genetic comparisons of the native resident population only included sheep born on Ram Mountain. Population‐wide genetic properties of Ram Mountain native residents were compared at two‐generation (13 years) intervals using a Wilcoxon signed rank test pairing the data by locus (Johnson, Bellinger, Toepfer, & Dunn, 2004). We used the same test to compare translocated individuals with native residents at Ram Mountain prior to translocations.

We used generalized linear (GLMM) and linear mixed models (LMM) to analyse the effect of outbreeding on fitness‐related traits in juveniles from 2003 to 2016. Models were fitted using the *lme4* package (Bates, Mächler, Bolker, & Walker, 2015). LMMs were computed for lamb mass at weaning, lamb summer mass gain, birthdate and yearling mass in September as response variables. We used a GLMM with binomial distribution for lamb survival to 1 year. Yearling survival could not be investigated with a GLMM due to low variance in survival and nonconvergence of models. A Fisher's exact test was thus used to compare survival of F_1_ and nonadmixed yearlings of each sex. We included maternal identity and year as random terms in all mixed models because some individuals were born to the same ewe in different years. Conformity of models to assumptions of independence, homoscedasticity and normality of residuals was assessed through visual inspection of residuals. Mixed models were run separately for each of the five responses variables. Predictors of interest were admixture (“Res”, “F_1_”, “F_2_”) and the interaction between sex and admixture (Rioux‐Paquette et al., 2011). In addition to the two predictors of interest and depending on the response variable, we controlled for birthdate, maternal mass and previous reproductive success, and season‐specific PDO. In all cases, continuous explanatory variables were standardized by centring and dividing by two standard deviations (Gelman, 2008). Standardization facilitated model convergence and interpretation of estimates (Schielzeth, 2010). Model selection followed a backward stepwise procedure to remove nonsignificant (*p* > 0.05) fixed effects (Crawley, 2012). Explained variance of selected models was quantified with marginal and conditional *R* ^2^ (Nakagawa & Schielzeth, 2013) (Supporting Information Table S2.3). Finally, using the “best” models, we carried out a post hoc analysis to investigate the effect of sex of the translocated parent on fitness‐related traits of F_1_ lambs (Supporting Information Appendix S3).

### 2.5 Population viability analysis

In addition to individual‐based models of juvenile fitness, we constructed two 4 x 4 age‐structured female‐based stochastic matrix models to simulate demographic consequences of genetic rescue (Supporting Information Appendix S4). Using 2003–2016 as reference years, we compared survival and fecundity in two scenarios. The first scenario was based upon estimated age‐specific survival and reproductive rates of nonadmixed native residents in 2003–2016 and simulated population trajectories in the absence of genetic rescue. The second scenario included survival and reproductive rates of both nonadmixed and admixed native residents and simulated dynamics with genetic rescue. We used 292 and 330 female‐years of survival and fecundity values to generate age‐specific bootstrapped (10,000 iterations) estimates of vital rates for the first and second scenario, respectively. Since information on admixed females aged >4 years old was scarce, we used vital rates for native resident adult females in both scenarios (Supporting Information Appendix S4). Using the *popbio* package (Stubben & Milligan, 2007), we simulated both scenarios for 50 years starting from the 2003 population size to estimate asymptotic population growth rate, population size and quasi‐extinction probability, defined as when the number of adult females declined to <10 (Boyce, 1992; Sibly & Hone, 2002) (Supporting Information Appendix S4).

## 3 RESULTS

### 3.1 Genetic decline

**Figure 1:**
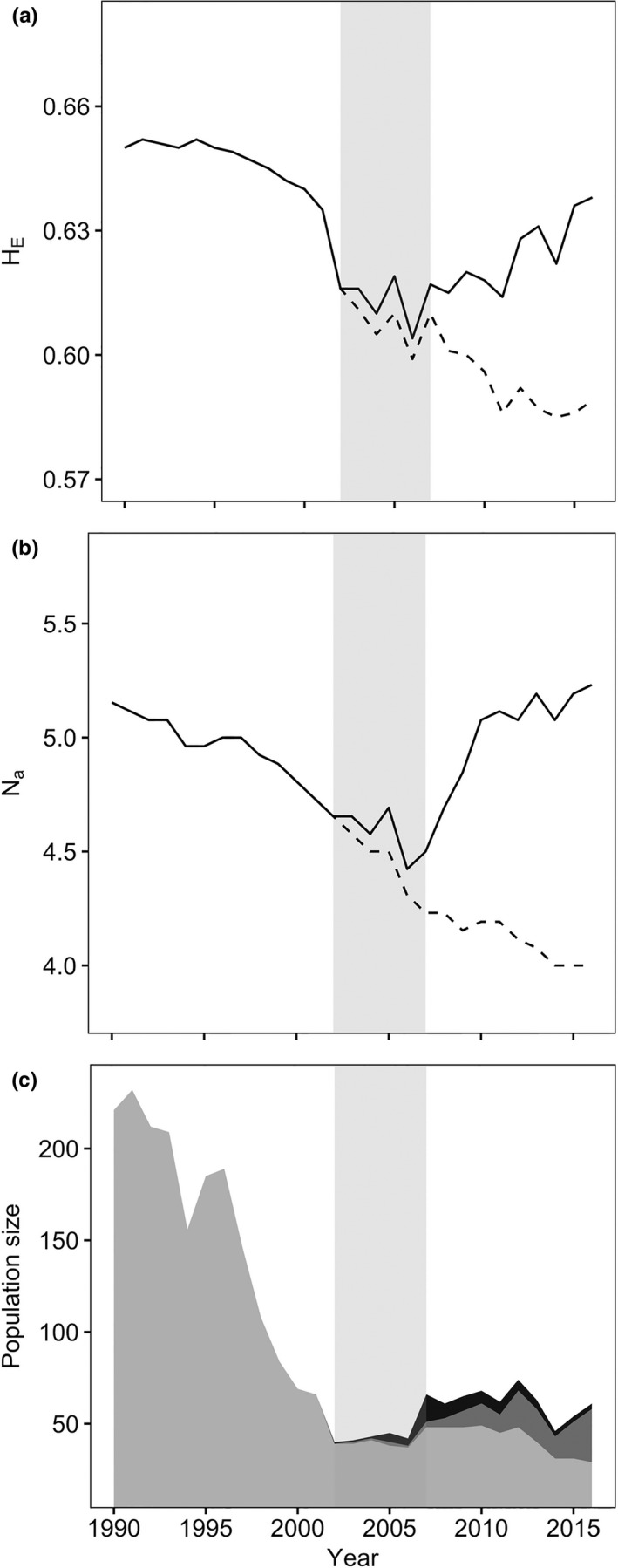
Genetic and demographic changes in the Ram Mountain bighorn sheep population, Alberta, Canada (1990–2016). (a) Population‐wide expected heterozygosity and (b) mean number of alleles per locus for the “native resident” group excluding translocated sheep (solid line) and the “nonadmixed native resident” group which only includes endemic Ram Mountain descendants (dashed line). (c) Population size with the number of nonadmixed (light grey), admixed (grey) and translocated (black) sheep. Vertical grey bars indicate the translocation period (2002–2007)

Over two generations, in 1990–2003, the Ram Mountain population declined from 221 to 40 sheep and lost 16 of 134 alleles from 26 monitored microsatellite loci. During this period, expected heterozygosity and number of alleles decreased by 6.2% and 11.3% (Table 2, Figure 1a,b), respectively. Population‐wide inbreeding (*F*_IS_), however, did not differ between 1990 and 2003 (Table 2). Genetic differentiation (*F*_ST_) was 0.015 (95% CI: 0.006–0.027) between the pre‐ and postdecline Ram Mountain native resident population. An additional four alleles present in 1990–2003 were lost before 2016 (Table 2), for a 14.9% decline in endemic resident alleles since 1990.

**Table 2:** Genetic properties at 26 polymorphic microsatellite loci in the Ram Mountain bighorn sheep population prebottleneck (1990), pretranslocation (2003) and post‐translocation (2016)

	1990 (*n* = 195)	2003 (*n* = 38)	2016 (*n* = 58)	*p*‐Value^a^	*p*‐Value^b^
*N*_a_	5.15	4.58	5.23	0.002	0.002
*A*_R_	4.35	4.21	4.53	0.043	0.012
*H*_E_	0.650	0.610	0.638	<0.001	0.040
*H*_O_	0.665	0.632	0.632	0.119	0.439
*F*_IS_	−0.023	−0.022	0.016	0.286	0.068

Notes

Comparisons are made at approximately two‐generation intervals. *p*‐Values are shown for declining^a^ (1990 vs. 2003) and recovering^b^ (2003 vs. 2016) periods. The 2003 sample in Tables 2 and 3 excludes the first admixed offspring born in 2003. The 2003 and 2016 samples exclude all translocated individuals. *H*_E_ and *H*_O_ did not differ significantly for a given time. *F*_IS_ slightly differed from zero in 1990 (95% CI: −0.0034 to −0.0419) but did not differ significantly from zero in 2003 and 2016.

### 3.2 Translocations

Of 26 bighorn sheep translocated in 2002–2007, only nine (35%) reproduced in the study population (Table 1). For translocated sheep that remained on Ram Mountain long enough to potentially reproduce, mean allelic richness and expected heterozygosity were significantly higher than for the 2003 nonadmixed native resident population (Table 3). Neither genetic population, however, showed signs of inbreeding (*F*_IS_; Table 3). Population genetic differentiation (*F*_ST_) between translocated individuals and the 2003 nonadmixed native residents was 0.127 (95% CI: 0.088–0.167), and both populations contained several private alleles (Table 3).

**Table 3:** Genetic properties at 26 polymorphic microsatellite loci of the pretranslocation (2003) Ram Mountain native resident bighorn sheep and translocated individuals (2002–2007)

	Pretranslocation Ram Mountain (*n* = 38)	Translocated individuals (*n* = 17)	*p*‐Value
*N*_a_	4.58	5.08	0.065
*A*_R_	4.21	5.06	0.003
Private alleles (%)	17 (14.3)	30 (22.7)	
*H*_E_	0.610	0.673	0.038
*H*_O_	0.632	0.693	0.127
*F*_IS_	−0.022	−0.002	0.114

Note

Private alleles are those not found in the compared sample.

### 3.3 Genetic restoration

Comparison of the pre‐ and post‐translocation native resident populations identified 21 alleles that introgressed from translocated sheep, or 70% of the private alleles found in translocated individuals (Table 3). The new alleles significantly increased genetic diversity over two generations (Table 2; Figure 1a,b). Mean number of alleles and allelic richness increased by 14.3% and 7.8%, respectively (Table 2). Expected heterozygosity increased by 4.6% and observed heterozygosity did not differ significantly between pre‐ and post‐translocation populations (Table 2). Genetic differentiation (*F*_ST_) was 0.022 (95% CI: 0.010–0.035) between the pre‐ and post‐translocation Ram Mountain native resident population. Genetic restoration began in 2006 and genetic diversity of the native resident group recovered to values measured in the predeclining (1990) population by 2016 (Figure 1a,b). Meanwhile, the genetic diversity of nonadmixed native residents continued to decline (Figure 1a,b).

### 3.4 Genetic rescue

**Figure 2:**
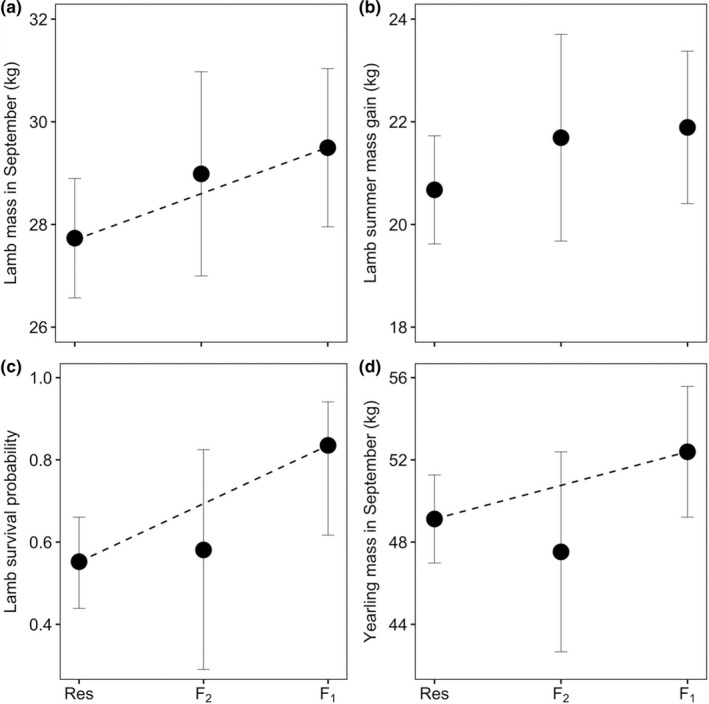
Fitness‐related traits of bighorn juveniles at Ram Mountain, Alberta, Canada (2003–2016) following population supplementation. “Res” indicates nonadmixed residents, “F_1_” those with one translocated parent (50% ancestry from translocated sheep) and “F_2_” those with one translocated grandparent (25% ancestry from translocated sheep). (a) Lamb mass in September (kg), (b) lamb summer mass gain (kg), (c) lamb survival to 1 year and (d) yearling mass in September (kg). Error bars represent 95% CIs. Dashed lines are shown for statistically significant (*p* < 0.05) effects. Data on males are presented for models with a significant effect of sex (see Supporting Information Table S2.2)

Observed increases in genetic diversity were accompanied by improvements in several fitness measures for F_1_ juveniles. At weaning, F_1_ lambs were 6.4% heavier than nonadmixed lambs (Table S2.2; Figure 2a,b). More importantly, survival to 1 year was 28.3% higher for F_1_ lambs compared with nonadmixed lambs (Supporting Information Table S2.2; Figure 2c). Admixture did not affect date of birth (Supporting Information Table S2.2). In contrast to earlier results (Rioux‐Paquette et al., 2011), lamb overwinter survival was not correlated with mass in September (*t*_98.911_ = −0.48, *p* = 0.63) or summer mass gain (*t*_66.681_ = −1.59, *p* = 0.12). The interaction between sex and admixture was not a significant predictor for any response variable for lambs or yearlings (Supporting Information Table S2.2). Males, however, gained more mass as lambs and were heavier in September both as lambs and yearlings (Supporting Information Table S2.2). F_2_ offspring did not show significant differences in fitness for any of the five traits tested (Supporting Information Table S2.2). For F_1_ lambs, sex of the translocated parent had no effect on most fitness‐related traits tested, but lambs sired by translocated males were 2.4 kg heavier at weaning than those born to translocated mothers (*β* = 2.358, *SE* = 0.885, *p* = 0.013, Supporting Information Table S3.2). Compared with nonadmixed yearlings, F_1_ yearlings were 7.3% heavier in September (Supporting Information Table S2.2, Figure 2d). Yearling survival to 2 years did not differ between nonadmixed and F_1_ for both males (Fisher's exact test, *p* = 0.333; Supporting Information Figure S2.1) and females (Fisher's exact test, *p* = 0.558; Supporting Information Figure S2.1). All F_1_ and F_2_ female yearlings survived to 2 years.

### 3.5 Population viability analysis

**Figure 3:**
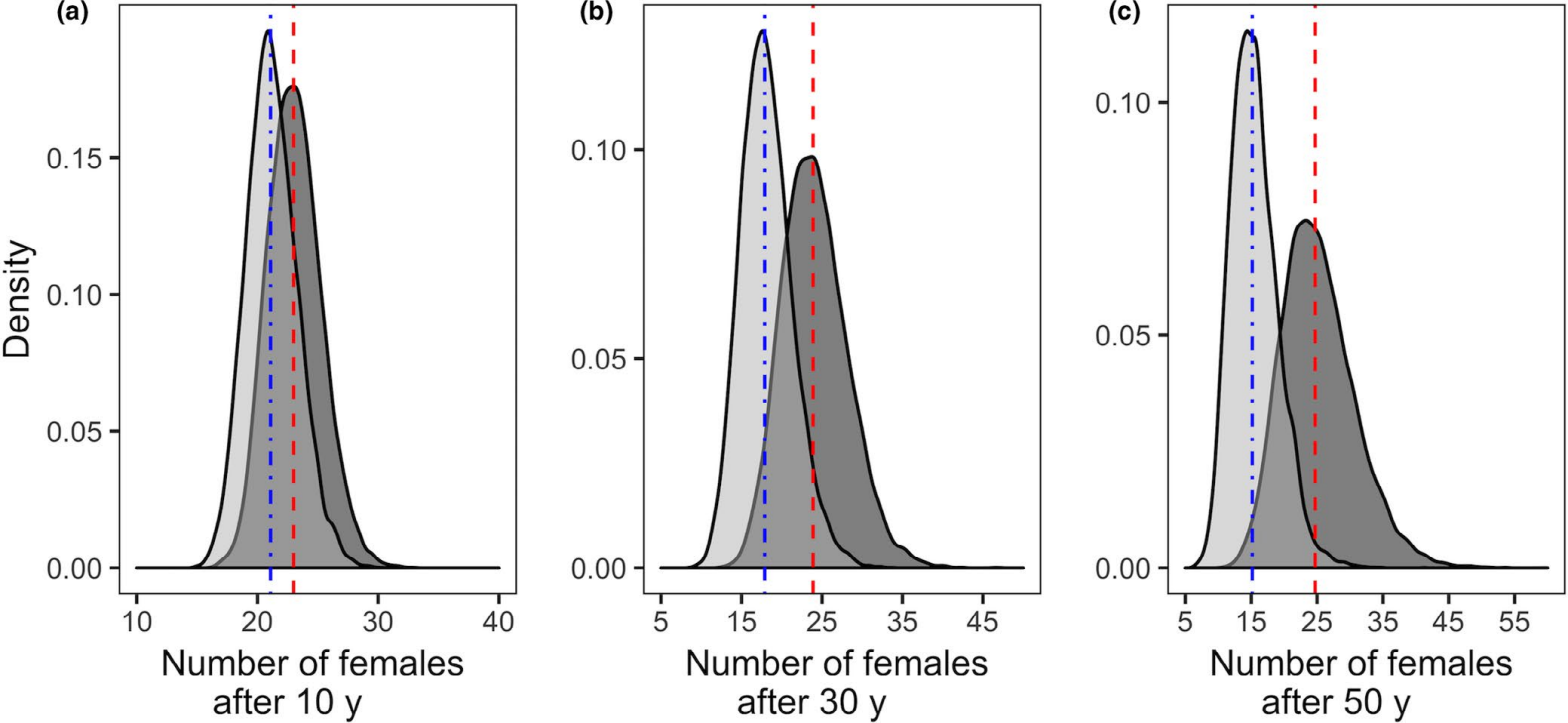
Contrasted simulated population sizes (number of females) after 10 (a), 30 (b) and 50 (c) years for scenarios with genetic rescue (dark grey) and without genetic rescue (light grey) based on 10,000 iterations. Dashed red and blue vertical lines represent median population sizes for the scenarios with and without genetic rescue, respectively. Simulation started at year zero with 23 females, the number alive at Ram Mountain in 2003. Significant differences between both scenarios are mostly observed after 30 years

Comparison of predicted populations for scenarios with and without genetic rescue suggested small differences in population growth. Asymptotic growth rate (*λ*) differed by 1.3% in scenarios with (*λ* = 1.004; 95% CI = 0.94–1.06) and without genetic rescue (*λ* = 0.991; 95% CI = 0.93–1.05) (Supporting Information Appendix S4). Predicted differences in population size over 10,000 iterations remained small in the first 10–20 years but increased after 30 years (Figure 3). The quasi‐extinction probabilities, defined as fewer than 10 adult females remaining in the population, were 0.9% and 44.6% after 50 years with and without genetic rescue, respectively (Supporting Information Figure S4.4). However, quasi‐extinction probability remained low for the first 10–20 years in both scenarios. See Supporting Information Appendix S4, for additional results.

### 3.6 Population recovery

Demographic recovery appeared to be ongoing in 2012 when the population size reached 74 sheep, an increase of 85% since 2003 (Figure 1c). High cougar predation in 2012–2013, however, led to a 38% decline. Predation ceased in 2014 and the population increased to 61 sheep in 2016. As of 2016, 50% of residents were admixed (Figure 1c). The expected proportion of introgressed alleles in the resident population was estimated at 19.1% based on individual ancestries derived from the population's pedigree. The population increased to 73 in 2017, excluding an additional nine sheep translocated in 2015 and their lambs (Poirier & Festa‐Bianchet, 2018), an 82.5% increase since 2003.

## 4 DISCUSSION

Prior to decline, genetic diversity at Ram Mountain was within expected values for bighorn sheep populations at similar latitudes ( Coltman, unpublished data). High predation following a density‐dependent decline led to a demographic bottleneck and population stagnation. The drastic decline of 83% in population size over two generations was followed by a significant decrease in genetic diversity as predicted by genetic drift (Nei et al., 1975). Temporal monitoring indicated a faster reduction in allelic diversity than in heterozygosity, the expected signature of a bottleneck (Maruyama & Fuerst, 1985; Nei et al., 1975). Contributions of a few translocated individuals, however, allowed genetic diversity to recover. Over two generations, introgression of new alleles allowed the population to reach greater genetic diversity than before the demographic decline. With nine translocated sheep reproducing in the local population of 40–60 sheep, there is no immediate evidence of genetic swamping of local genes, based on population‐wide proportion of migrant alleles. Continued monitoring of the population, and especially of functional genes (Miller, Festa‐Bianchet, & Coltman, 2018), will give further insight on the genetic consequences of the translocations. Greater standing genetic variation should increase the potential for genetic and evolutionary rescue (Barrett & Hendry, 2012; Whiteley et al., 2015).

At Ram Mountain, prior to translocations, juvenile recruitment was the dominant demographic parameter in periods of population stagnation and increase (Coulson, Gaillard, & Festa‐Bianchet, 2005). Consequently, poor survival of inbred female lambs (Rioux‐Paquette et al., 2011) and low numbers of reproductive adults following the demographic bottleneck threatened the persistence of this population. Additionally, threats of stochastic predation events (Festa‐Bianchet et al., 2006) further increased risks of population decline. Outbreeding of resident sheep with translocated individuals led to a substantial increase in survival for F_1_ lambs providing an opportunity for recovery. We suggest that the increase in population size observed in 2015–2017 was partly attributable to increased fitness of F_1_ juveniles. Our comparison of female‐based demographic scenarios with and without genetic rescue suggests that even a small input of outbred sheep favours population persistence. Those demographic scenarios, however, should be interpreted with caution because they used the same vital rates for adult females and simulated a fixed proportion of admixed individuals in the population over time (Supporting Information Appendix S4). Beneficial effects of genetic rescue are expected to stabilize by the F_3_ generation and to remain at that level subsequently, unless the population becomes inbred again (Frankham, 2016). Moreover, the proportion of admixed sheep and the fraction of migrant alleles should increase over time as translocated individuals were still contributing to the population in 2017. Consequently, our population viability simulations may minimize actual differences between scenarios with and without genetic rescue. Nonetheless, the higher survival of admixed juveniles should lead to more reproductive adults, which have high survival and are likely to contribute to population growth for many years (Gaillard et al., 1998; Gaillard, Festa‐Bianchet, Yoccoz, Loison, & Toïgo, 2000). Hogg, Forbes, Steele, and Luikart (2006) found that outbreeding was associated with greater annual reproductive success and increased survival for both male and female adult bighorn sheep. We found strong evidence for increased fitness in juveniles, providing further support for genetic rescue in naturally occurring populations. Despite sex‐differential effects of inbreeding in the local population (Rioux‐Paquette et al., 2011), outbreeding did not affect the sexes differently in any of the fitness‐related traits tested. Furthermore, F_1_ lambs of both sexes had higher fitness compared with nonadmixed lambs regardless of the sex of the translocated parent. Contrary to our expectations, however, we did not find evidence for increased fitness in F_2_ juveniles. Smaller sample sizes for F_2_ and lack of statistical power may explain these different results. Further investigation will seek to identify the mechanisms behind the genetic rescue of Ram Mountain where significant increases in F_1_ fitness were observed despite modest decline in genetic diversity. Increased fitness following outbreeding may have occurred through a reduction in inbreeding or adaptive evolution (Whiteley et al., 2015).

The potential for outbreeding depression is a primary concern with translocations and is supported by theory (Edmands, 2007). Recent theoretical and empirical examples, however, suggest that naturally or artificially establishing gene flow among populations will usually increase fitness (Frankham et al., 2011; Heber et al., 2013; Weeks et al., 2011). In this study, genetic differentiation between resident and translocated sheep was significant (12.7%). Despite its relative isolation and genetic differentiation from translocated individuals, the Ram Mountain population appeared to recover both at the genetic and demographic levels following translocations. We found no evidence for outbreeding depression, with admixture in lambs and yearlings having positive or null effects in all fitness‐related traits tested. Continued genetic monitoring of the population will assess potential swamping of locally adaptive variants in the long term. We suspect, however, that without the translocations, loss of genetic diversity would have continued through increased genetic drift as the population continued to decline (Figure 1).

Translocations of bighorn sheep are frequently used for conservation of this species (Brewer et al., 2014). Our results may guide wildlife managers interested in increasing genetic diversity of isolated populations and enhance fitness of population suffering from inbreeding depression. We point out, however, that clear evidence of inbreeding depression at Ram Mountain justified this intervention, similarly to findings for Florida panthers (Hedrick & Fredrickson, 2010; Johnson et al., 2010). Trophy hunting of bighorn sheep is a major socio‐economic activity (Festa‐Bianchet & Lee, 2009). We caution against broad‐scale translocations simply to increase trophy quality, especially given threats associated with transmission of pathogens and the lack of cross‐strain immunity to infectious disease among bighorn sheep populations (Cassirer, Manlove, Plowright, & Besser, 2017).

Given the current rate of habitat loss and human‐induced environmental changes, many populations may not be able to adapt or persist. Species with long generation times, such as bighorn sheep, are the least likely to be rescued by evolution (Vander Wal et al., 2012). Consequently, understanding how natural or artificial immigration can reduce or enhance the opportunity for adaptation and rescue in long‐lived species is currently of significant importance (Carlson et al., 2014; Tallmon et al., 2004; Whiteley et al., 2015). Individual‐based long‐term studies that monitor populations will provide key insights about the consequences of both decline and recovery at the genetic, demographic and phenotypic levels for wild populations. Here, we demonstrated how the contribution of a few translocated individuals can substantially affect population genetic properties, individual fitness and demographic rates in a small population suffering from inbreeding depression. Our results suggest that translocations of large mammals and artificial gene flow may be one promising way to mitigate the effect of human‐driven environmental changes on wild population and allow large vertebrate populations to persist.

## ETHICAL APPROVAL

The study was authorized by the Université de Sherbrooke Animal Care Committee, affiliated with the Canadian Council on Animal Care, protocols MFB2006‐01 and MFB2014‐01.

## CONFLICT OF INTERESTS

The authors declare no conflict of interests.

## AUTHORS’ CONTRIBUTIONS

M.A.P. designed the study, conducted statistical analyses and wrote the first draft of the manuscript with input from M.F.B. and F.P.; D.C. performed molecular analyses and paternity assignments; M.A.P., M.F.B. and J.J. collected field data; and J.J. supervised sheep translocations. All authors contributed to the writing and revision of the manuscript.

## Data Availability

Data available from the Dryad Digital Repository: https://doi.org/10.5061/dryad.n8v973b.
