# Supplementary material for: Genetic decline, restoration and rescue of an isolated ungulate population
Source: Evol Appl. 2018 Nov 27;12(7):1318–28. doi: 10.1111/eva.12706 (PMC6691324; doi:10.1111/eva.12706)
Supplement: Supplementary file 1 [file EVA-12-1318-s001.docx]

Supplementary Material

*Genetic decline, restoration and rescue of an isolated ungulate population*

*Marc-Antoine Poirier, David W. Coltman, Fanie Pelletier, Jon Jorgenson and Marco Festa-Bianchet*

*Supplementary Material S1: Additional information on past and present genetic connectivity, habitat quality, and population size for both the source and destination populations. Also includes details on the selection of translocated individuals.*

*S1.1 Bighorn sheep in North America*

Bighorn sheep recolonized the Canadian Rockies after the last glaciation about 10 000 years ago. Generally, Canadian populations are less genetically diverse than those in the USA who survived in refuges south of the ice sheet (Luikart & Allendorf, 1996). Following European settlement, bighorn sheep populations declined substantially throughout North America and are now found in a fraction of the habitat they occupied in the 19th century (Toweill & Geist, 1999). Habitat loss and infectious diseases (Cassirer & Sinclair, 2007; Manlove, Cassirer, Cross, Plowright, & Hudson, 2014) have led several wild populations to isolation. In the last century alone, more than 20 000 sheep have been translocated for reintroduction programs or supplementation of wild populations (Brewer et al., 2014).

*S1.2 Ram Mountain and Cadomin populations*

Ram Mountain, Alberta (52 ° N, 115 ° W, elevation 1080 to 2170 m), is a mountainous outcrop located 30 km east of the Canadian Rockies. Bighorn sheep occupy approximately 38 km^2^ of this habitat characterized by alpine and subalpine grasslands. The population is geographically isolated and immigration is rare, with the exception of a few individuals from nearby Shunda Mountain (approx. 4 km). The alternative source of immigrants, in the main Rocky Mountain range, is over 30 km away and separated by coniferous forests, an unsuitable habitat for bighorn sheep. In 2002-2007, low and stagnant population size (40-45 sheep) justified the translocation of sheep from another population to Ram Mountain.

The source population at Cadomin (53° N, 117° W) lies 130 km northwest of Ram Mountain and is estimated to number more than 500 sheep. Bighorn sheep at Cadomin make heavy use of reclaimed coal mine habitat seeded with domestic grasses and native vegetation, arguably of higher quality and quantity than what is available on Ram Mountain (MacCullum & Geist, 1992).

As bighorn sheep recolonized the Canadian Rockies moving south-north following deglaciation, both Ram Mountain and Cadomin populations likely had the same source. The genetic distance between the Ram Mountain and Cadomin population is estimated at 6% (Fst = 0.06). This distance is based on our unpublished population genetic data and is considered “moderate” compared to other sheep populations in Alberta. Genetic diversity at Cadomin is within the expected values for bighorn sheep populations at similar latitudes (D. Coltman, unpublished data).

*S1.3 Sheep selected for translocation*

Translocated individuals were chosen at random from the source population. The only selection criterion was to avoid old animals to insure relocated individuals could contribute to the destination population for many years. Individuals translocated before 2007 were caught with a drop net. Since most animals older than 2 years left Ram Mountain following their relocation in 2004-2005, those translocated in 2007 were all yearlings (Table 1; main text) caught with a dart gun. Using mass measurements collected a few months following their relocation, we previously showed that the mass of translocated yearlings did not differ from that of Ram Mountain resident yearlings (Poirier & Festa-Bianchet, 2018), suggesting that translocated individuals were in similar condition to residents of the destination population.

### S2. Genetic rescue analysis (supplementary material)

###### **Table S2.1** Predictor variables used in models presented in Table S2.2.

| Predictors | Description |
| --- | --- |
| Admixture | Lamb or yearling admixture status (categorical) |
| Sex | Lamb or yearling sex |
| BD | Lamb date of birth |
| MMS | Maternal mass in September |
| MMPS | Maternal mass the previous September |
| PRS | Maternal previous reproductive success |
| nwPDO | Next winter PDO |
| sPDO | Summer PDO |
| rPDO | Rut PDO |

**Table S2.2.** Parameter estimates of the best model for fitness-related traits in lambs and yearling bighorn sheep at Ram Mountain, Canada (2003–2016). Non-admixed (residents of endemic ancestry) and females were used as reference. All models included maternal identity and year as random terms. All continuous explanatory variables were standardized.

|  |  | | estimate | s.e. | *T*-^a^ or  *Z*-value^b^ | *p*-value |
| --- | --- | --- | --- | --- | --- | --- |
| Birthdate^a^ (*n*=166) | | |  |  |  |  |
| Full model: Admix + Sex + MMPS + PRS + rPDO + Admix:Sex | | | | | | |
| Final model | | | | | | |
| Intercept |  | | 151.737 | 19.992 | 76.180 | **< 0.001** |
| Admixture | F_1_ | | 2.451 | 2.880 | 0.850 | 0.397 |
|  | F_2_ | | 6.016 | 3.619 | 1.660 | 0.103 |
| Maternal mass previous September | | | -6.179 | 2.238 | 2.760 | **0.008** |
|  |  | |  |  |  |  |
| Lamb summer mass gain^a^ (*n*=129) | | |  |  |  |  |
| Full model: Admix + Sex + BD + MMS + sPDO + Admix:Sex | | | | | | |
| Final model | | | | | | |
| Intercept |  | | 19.237 | 0.543 | 35.441 | **< 0.001** |
| Admixture | F_1_ | | 1.217 | 0.670 | 1.820 | 0.072 |
|  | F_2_ | | 1.016 | 0.975 | 1.040 | 0.300 |
| Lamb sex |  | | 1.437 | 0.442 | 3.250 | **0.002** |
| Birthdate |  | | -2.248 | 0.488 | 4.610 | **< 0.001** |
| Maternal mass in September | | | 2.400 | 0.559 | 4.300 | **< 0.001** |
|  |  | |  |  |  |  |
| Lamb mass in September^a^ (*n*=140) | | |  |  |  |  |
| Full model: Admix + Sex + BD + MMS + sPDO + Admix:Sex | | | | | | |
| Final model | | |  |  |  |  |
| Intercept |  | | 25.975 | 0.595 | 43.669 | **< 0.001** |
| Admixture | F_1_ | | 1.253 | 0.947 | 1.323 | **0.010** |
|  | F_2_ | | 1.763 | 0.674 | 2.615 | 0.189 |
| Lamb sex |  | | 1.758 | 0.455 | 3.862 | **< 0.001** |
| Birthdate |  | | -6.803 | 0.496 | 13.719 | **< 0.001** |
| Maternal mass in September | | | 3.587 | 0.573 | 6.259 | **< 0.001** |
|  |  | |  |  |  |  |
| Lamb survival to 1 year^b^ (*n*=149) | | |  |  |  |  |
| Full model: Admix + Sex + BD + MMS + nwPDO + Admix:Sex | | | | | | |
| Final model | | |  |  |  |  |
| Intercept |  | | 0.210 | 0.233 | 0.902 | 0.367 |
| Admixture | F_1_ | | 1.413 | 0.609 | 2.320 | **0.020** |
|  | F_2_ | | 0.117 | 0.648 | 0.180 | 0.857 |
|  |  | |  |  |  |  |
| Yearling mass in September^a^ (*n*=84) | | | |  |  |  |
| Full model: Admix + Sex + sPDO + Admix:Sex | | | | | | |
| Final model | | | | | | |
| Intercept |  | | 43.854 | 1.048 | 41.851 | **< 0.001** |
| Admixture | F_1_ | | 3.268 | 1.508 | 2.167 | **0.033** |
|  | F_2_ | | -1.601 | 2.462 | 0.650 | 0.518 |
| Yearling sex | |  | 5.270 | 1.070 | 4.927 | **< 0.001** |
| Summer PDO | | | 2.435 | 1.104 | 2.205 | **0.031** |

^a^ Analyses used linear mixed model.

^b^ Analysis used generalized linear mixed model with a binomial distribution.

**Table S2.3.** Marginal and Conditional R^2^ of the final (‘best’) models for tested fitness-related traits in bighorn juveniles at Ram Mountain, Alberta, Canada (2003-2016). Models are described in Table S2.2.

|  | *n* | *Marginal R^2^* | *Conditional R^2^* | Family |
| --- | --- | --- | --- | --- |
| Lamb birthdate | 166 | 0.077 | 0.376 | Gaussian |
| Lamb summer mass gain | 129 | 0.325 | 0.592 | Gaussian |
| Lamb mass in September | 140 | 0.630 | 0.782 | Gaussian |
| Lamb survival to 1 year | 149 | 0.069 | 0.125 | Binomial |
| Yearling mass in September | 89 | 0.218 | 0.583 | Gaussian |


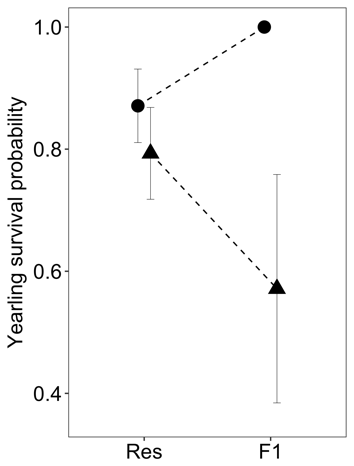


**Figure S2.1.** Survival to 2 years for non-admixed resident (Res) and F_1_ resident (F_1_) yearlings at Ram Mountain, Alberta, Canada (2003-2016). Males (*n*=36) are represented by filled triangles and females (*n*=42) by filled circles. Error bars represent standard error (±se). All F_1_ yearling females survived to 2 years. All F_2_ and F_3_ yearling females (*n*=6) also survived to 2 years (not shown in figure).

### S3. Effect of the sex of the translocated parent on first generation lambs

###### **Table S3.1** Predictor variables used in models shown in Table S3.2.

| Predictors | Description |
| --- | --- |
| Psex | Sex of translocated parent of F_1_ lambs |
| Sex | Lamb sex |
| BD | Lamb date of birth |
| MMS | Maternal mass in September |
| MMPS | Maternal mass the previous September |

###### **Table S3.2.** Parameter estimates of the best model for four fitness-related traits of F_1_ lambs at Ram Mountain, Alberta, Canada (2003–2016). Females were used as reference for both ‘sex of the translocated parent’ and ‘lamb sex’. All models included maternal identity and year as random terms. All continuous explanatory variables were standardized.

|  |  | estimate | s.e. | *T*-^a^ or *Z*-value^b^ | *p*-value |
| --- | --- | --- | --- | --- | --- |
| Birthdate^a^ (*n*=28) | |  |  |  |  |
| Full model: Psex + Sex + MMPS + Psex:Sex | | | |  |  |
| Final model |  |  |  |  |  |
| Intercept |  | 151.500 | 3.253 | 46.580 | **< 0.001** |
| Sex of translocated parent | | 2.071 | 4.600 | 0.450 | 0.656 |
|  |  |  |  |  |  |
| Lamb summer mass gain^a^ (*n*=23) | | |  |  |  |
| Full model: Psex + Sex + BD + MMS + Psex:Sex | | | |  |  |
| Final model |  |  |  |  |  |
| Intercept |  | 20.610 | 0.703 | 29.320 | **< 0.001** |
| Sex of translocated parent | | 1.098 | 0.767 | 1.432 | 0.190 |
| Birthdate |  | -1.841 | 0.598 | 3.081 | **0.007** |
|  |  |  |  |  |  |
| Lamb mass in September^a^ (*n*=26) | | |  |  |  |
| Full model: Psex + Sex + BD + MMS + Psex:Sex | | | |  |  |
| Final model |  |  |  |  |  |
| Intercept |  | 25.929 | 0.699 | 37.091 | **< 0.001** |
| Sex of translocated parent | | 2.358 | 0.885 | 2.665 | **0.013** |
| Lamb sex |  | 2.516 | 0.900 | 2.796 | **0.010** |
| Birthdate |  | -7.854 | 0.842 | 9.332 | **< 0.001** |
| Maternal mass in fall | | 2.627 | 0.940 | 2.796 | **0.010** |
|  |  |  |  |  |  |
| Lamb survival to 1 year^b^ (*n*=28) | | |  |  |  |
| Full model: Psex + Sex + Psex:Sex | | |  |  |  |
| Final model |  |  |  |  |  |
| Intercept |  | 2.138 | 1.406 | 1.520 | 0.128 |
| Sex of translocated parent | | -0.589 | 1.235 | 0.477 | 0.633 |

^a^ Analyses used linear mixed model.

^b^ Analysis used generalized linear mixed model with a binomial distribution.

### S4. Population viability analysis

We constructed two female-based 4x4 Leslie matrices (Figure S4.1) to investigate the demographic effects of genetic rescue in the Ram Mountain population. Bighorn sheep survival and fecundity is adequately represented by four age classes for females (Jorgenson, Festa-Bianchet, Gaillard, & Wishart, 1997). We used 2003-2016 survival and reproductive success of known-aged females (Table S4.1) to compute bootstrapped estimates of vital rates for lambs, yearlings, prime-aged (2-7 y.o) and senescent (8+ y.o.) females. The scenarios differed only in juvenile vital rates for two reasons. First, no data were yet available for admixed (i.e. outbred) senescent females because they were too young or because they died before reaching 8 years old. Second, young admixed prime-aged females (3-4 y.o., n=25 female-years) were over-represented compared with older admixed prime-aged females (6-7 y.o., n=8 female-years), giving an inaccurate picture of vital rates for this age-class. Therefore, our results show the apparent population dynamic consequences of a small input of outbred sheep that only have increased juvenile survival (Table S4.1). Hogg et al. 2006 (Hogg, Forbes, Steele, & Luikart, 2006) previously found that outbreeding was associated with greater annual reproductive success and increased survival for both male and female adult bighorn sheep in the National Bison Range population, Montana, USA. Nonetheless, we used resident adult female vital rates in both scenarios because of limited data for admixed adults at Ram Mountain. This insured that our analysis would not overestimate beneficial effects of genetic rescue on population growth.

We included in our models a carrying capacity, *k*, limiting simulated populations to 100 females aged 2 years and older based on significant density-dependence effects observed in the population (Festa-Bianchet, Gaillard, & Côté, 2003). Our models did not take into account temporal variation in population-wide outbreeding/inbreeding frequencies. In addition, the genetic rescue scenario did not include the strictly demographic contribution of translocated females which increased the number of individuals in the population even in the absence of beneficial effects of outbreeding on fitness. For these reasons, our population viability analysis most likely underestimates the demographic effects of genetic rescue and translocations at Ram Mountain.

Demographic parameters were extracted from scenario-based population matrices using 10 000 bootstrapped estimates of age class-specific survival and fecundity (Table S4.2). Our 10 000 estimates per demographic parameter per age class yielded 10 000 different population matrices for which we measured asymptotic growth rate (λ), mean population size and quasi-extinction probability, defined as fewer than 10 adult females remaining in the population, over a 50-year period. The matrices were used to extract the dominant eigenvalue, i.e., the asymptotic population growth rate (Sibly & Hone, 2002) for each scenario using the ‘popbio’ package (Stubben & Milligan, 2007) in R v. 3.3.1 (R Development Core Team, 2015) (Figure S4.2). Starting from 2003 Ram Mountain population numbers, we then simulated 10 000 populations over 50 years to estimate mean predicted population size (Figure 3A; main text). Finally, to estimate quasi-extinction probabilities in both scenarios, we reran the population models with an added function that counted populations for which the number of females aged 2 years and older dropped below 10. We then estimated, out of 10 000 iterations, the proportion of populations that went ‘quasi-extinct’ over 50 years (Figure S4.4).


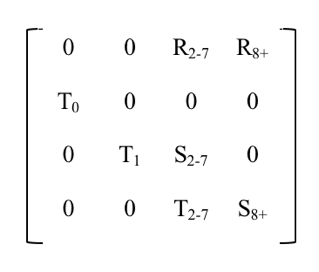


**Figure S4.1.** Female-based Leslie matrix where T_0_ is transition from lamb to yearling (i.e. survival from weaning to next summer), T_1_ is transition from yearling to prime-aged (2-7 years old), T_2-7_ is transition from prime-aged to senescent (8+ years old), S_2-7_ is prime-aged yearly survival, S_8+_ is senescent yearly survival, R_2-7_ is prime-aged reproductive success (i.e. weaned a female), and R_8+_ is senescent reproductive success (i.e. weaned a female).

**Table S4.1.** Sample sizes (female-years) available to estimate vital rates for non-admixed residents (total sample used for the scenario *without* genetic rescue), admixed residents (including F_1_, F_2_ and F_3_) and all residents (juvenile sample used for the scenario *with* genetic rescue) based on data collected in 2003-2016 at Ram Mountain, Alberta, Canada.

|  | Non-Admixed | Admixed | All Residents  (Adm. & Non-Adm.) |
| --- | --- | --- | --- |
| Lambs | 45 | 24 | 69 |
| Yearlings | 32 | 14 | 46 |
| 2-7 y.o. | 139 | 38 | 177 |
| 8+ y.o. | 76 | 0 | 76 |

**Table S4.2.** Estimated mean female vital rates in 2003-2016 at Ram Mountain, Alberta, Canada for scenarios *without* (left) and *with* (right) genetic rescue. Vital rates for adult females are the same in both scenarios. 95 Cis were derived from 10 000 bootstrapped estimates.

|  | Without genetic rescue | | | With genetic rescue | | |
| --- | --- | --- | --- | --- | --- | --- |
|  | Mean estimate | 95% CI | | Mean  estimate | 95% CI | |
|  |  | Lower | Upper |  | Lower | Upper |
| Survival |  |  |  |  |  |  |
| T_0_ | 0.733 | 0.600 | 0.867 | 0.754 | 0.652 | 0.855 |
| T_1_ | 0.844 | 0.718 | 0.969 | 0.891 | 0.804 | 0.978 |
| T_2-7_ | 0.108 | 0.056 | 0.159 | 0.108 | 0.056 | 0.159 |
| S_2-7_ | 0.770 | 0.698 | 0.835 | 0. 770 | 0. 698 | 0. 835 |
| S_8+_ | 0.776 | 0.684 | 0.868 | 0.776 | 0. 684 | 0. 868 |
| Recruitment |  |  |  |  |  |  |
| R_2-7_ | 0.223 | 0.158 | 0.295 | 0.223 | 0. 158 | 0. 295 |
| R_8+_ | 0.263 | 0.171 | 0.368 | 0.263 | 0. 171 | 0. 368 |


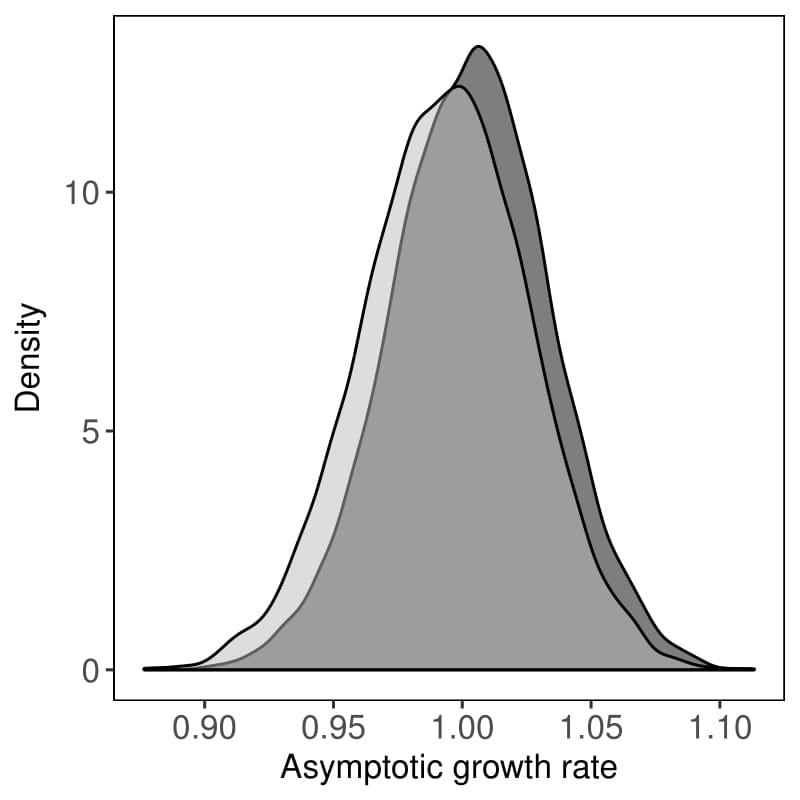


**Figure S4.2.** Contrasted asymptotic growth rates (λ) between two population scenarios (dark grey; *with* genetic rescue and light grey; *without* genetic rescue) at Ram Mountain, Alberta, Canada. Demographic parameters were extracted from scenario-based population matrices using bootstrapped estimates of age class-specific survival and fecundity (10 000 estimates per demographic parameter per age class, yielding 10 000 different population matrices).


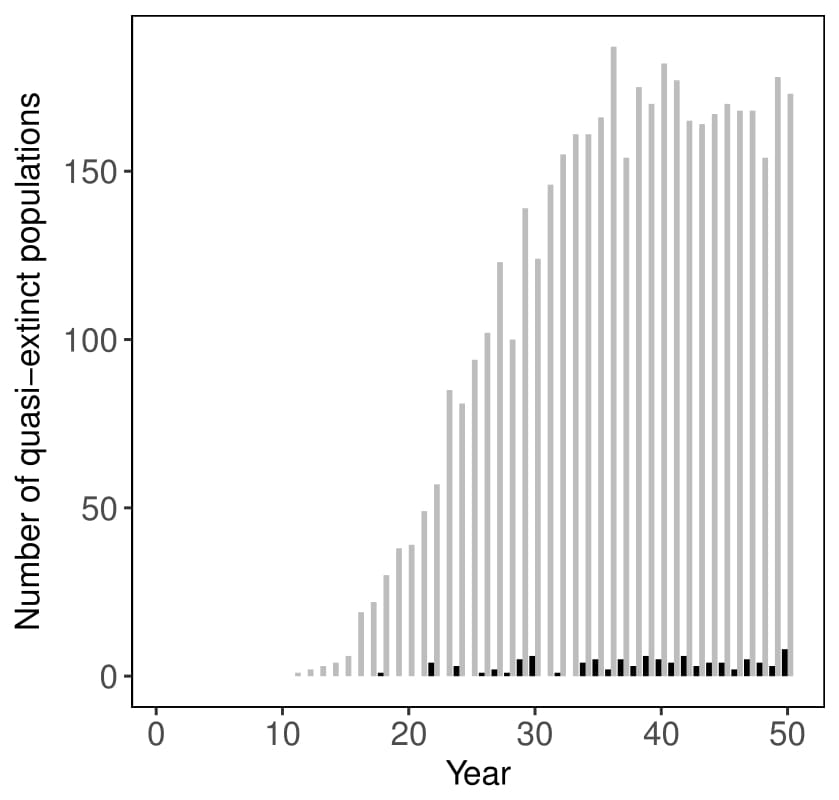


**Figure S4.3.** Number of quasi-extinct populations (number of females aged 2 years and older <10) over 50 years *with* (black bars) and *without* genetic rescue (grey bars) based on 10 000 iterations. For both scenarios, few simulated populations went quasi-extinct in the first 20 years (i.e. 2003-2023). About 11.8% and 44.6% of populations of the 10 000 simulated populations were quasi-extinct in the scenario *without* genetic rescue after 30 and 50 years, respectively. In contrast, only 0.9% of populations were quasi-extinct after 50 years in the scenario *with* genetic rescue.


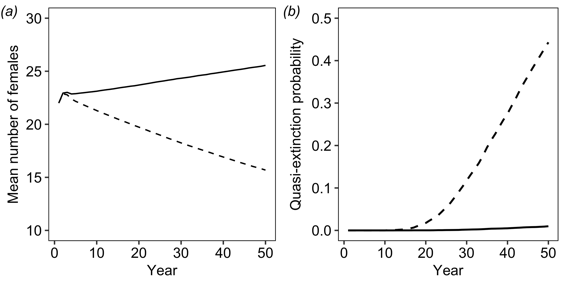


**Figure S4.4.** Contrasted (a) mean population size (number of females) and (b) quasi-extinction probability over 50 years between two simulated population scenarios (filled lines; *with* genetic rescue and dashed lines; *without* genetic rescue) at Ram Mountain, Alberta, Canada. Demographic parameters were extracted from scenario-based population matrices using bootstrapped estimates of age class-specific survival and fecundity (10 000 estimates per demographic parameter per age class, yielding 10 000 different population matrices). Simulation started with the 2003 Ram Mountain population numbers (year zero).

**References**

Brewer, C. E., Bleich, V. C., Hosch-Hebdon, T., McWhirter, D., Rominger, E., Wagner, M., … Foster, J. (2014). *Bighorn Sheep : Conservation Challenges and Management Strategies for the 21st Century*. Cheyenne, Wyoming, USA: Wild Sheep Working Group, Western Association of Fish and Wildlife Agencies.

Cassirer, E. F., & Sinclair, A. R. E. (2007). Dynamics of Pneumonia in a Bighorn Sheep Metapopulation. *Journal of Wildlife Management*, *71*(4), 1080–1088. https://doi.org/10.2193/2006-002

Festa-Bianchet, M., Gaillard, J. M., & Côté, S. (2003). Variable age structure and apparent density-dependence in survival of adult ungulates. *Journal of Animal Ecology*, *72*(1987), 640–649. Retrieved from http://www.jstor.org/view/00218790/ap060004/06a00130/0

Hogg, J. T., Forbes, S. H., Steele, B. M., & Luikart, G. (2006). Genetic rescue of an insular population of large mammals. *Proceedings of the Royal Society B: Biological Sciences*, *273*(1593), 1491–1499. https://doi.org/10.1098/rspb.2006.3477

Jorgenson, J. T., Festa-Bianchet, M., Gaillard, J.-M., & Wishart, W. D. (1997). Effects of age, sex, disease, and density on survival of bighorn sheep. *Ecology*, *78*(4), 1019–1032. https://doi.org/10.1890/0012-9658(1997)078[1019:EOASDA]2.0.CO;2

Luikart, G., & Allendorf, F. W. (1996). Mitochondrial-DNA Variation and Genetic-Population Structure in Rocky Mountain Bighorn Sheep (Ovis canadensis canadensis). *Journal of Mammalogy*, *77*(1), 109–123. https://doi.org/Doi 10.2307/1382713

MacCullum, B. N., & Geist, V. (1992). Mountain restoration: Soil and surface wildlife habitat. *GeoJournal*, *27*(1), 23–46. https://doi.org/10.1007/BF00150633

Manlove, K. R., Cassirer, E. F., Cross, P. C., Plowright, R. K., & Hudson, P. J. (2014). Costs and benefits of group living with disease : a case study of pneumonia in bighorn lambs ( Ovis canadensis ). *Proceedings of the Royal Society B: Biological Sciences*, *281*(November), 20142331.

Poirier, M.-A., & Festa-Bianchet, M. (2018). Social integration and acclimation of translocated bighorn sheep (Ovis canadensis). *Biological Conservation*, *218*, 1–9. https://doi.org/10.1016/J.BIOCON.2017.11.031

R Development Core Team, R. (2015). R: A Language and Environment for Statistical Computing. (R. D. C. Team, Ed.), *R Foundation for Statistical Computing*. R Foundation for Statistical Computing. https://doi.org/10.1007/978-3-540-74686-7

Sibly, R. M., & Hone, J. (2002). Population growth rate and its determinants: an overview. *Philosophical Transactions of the Royal Society B: Biological Sciences*, *357*(1425), 1153–1170. https://doi.org/10.1098/rstb.2002.1117

Stubben, C., & Milligan, B. (2007). Estimating and analyzing demographic models using the popbio package in R. *Journal Of Statistical Software*, *22*(11), 1–23. https://doi.org/10.18637/jss.v022.i11

Toweill, D. E., & Geist, V. (1999). *Return of royalty: wild sheep of North America*. Missoula. Montana, USA.: Boone & Crockett Club.
